# Intelligent Control and Digital Twins for Industry 4.0

**DOI:** 10.3390/s23084036

**Published:** 2023-04-17

**Authors:** Aleksei Tepljakov

**Affiliations:** Department of Computer Systems, Tallinn University of Technology, 12618 Tallinn, Estonia; aleksei.tepljakov@taltech.ee

One of the prominent features of the Fourth Industrial Revolution—frequently referred to as *Industry 4.0*—is “[that it is based on] cyber-physical production systems [and] the merging of real and virtual worlds” [1]. Furthermore, the path to achieving Industry 4.0’s goals involves the creation and implementation of advanced automation technologies, which are connected to the design of control systems in a broader sense [2]. Consequently, the present Special Issue is devoted to *intelligent control systems* as part of the rapidly evolving landscape of Industry 4.0 towards the next iteration known as *Industry 5.0*. Moreover, this Special Issue places importance on an additional crucial component, namely, *digital twins*. The five-dimensional model for digital twins, as introduced in [3], includes the real and the virtual entity, data storage, connections between these components, and a service layer and has demonstrated its significant potential through successful implementation in various fields in the industry. For this reason, the digital twin framework can be argued to have a considerable impact on the progress of Industry 4.0’s global rollout.

In fact, the digital twin framework streamlines the process of designing and developing intelligent control systems. By utilizing the five-dimensional model, a virtual representation of a real-life system or process is created, which consists of two fundamental components: a mathematical model or description and a tangible visual representation. The necessity for a mathematical description of the system brings us back to the fundamental concept of control systems, while the visual representation, in the most ideal situation, can also be interacted with in a manner that closely resembles real-life interaction. From this perspective, the visualization becomes endowed with more features, leading to an immersive experience for the end user, who can also meaningfully interact with it and thus achieve the desired results.

The communication and data storage components of digital twins are important as well because they facilitate the synchronization of parameters between the real-life system and its virtual counterpart. This aspect can be considered as part of the Internet-of-Things (IoT) landscape, and therefore, it is an additional area of interest for the present Special Issue. Additionally, problems related to data processing and analysis are also of interest, as these processes underpin the digital twin technology. An evident fact is that the development of intelligent control systems typically depends on an approach that utilizes data, which is true for data-driven applications in the domain of computational intelligence in general.

Let us now consider some articles already published in the present issue tackling the topics outlined above in various ways.

An important Industry 4.0 related concept called Maintenance 4.0 has been addressed in [4]. The article provides a systematic literature review and meta-analysis of Maintenance 4.0 in different application areas by analyzing 214 recent papers and assessing publication trends. The authors also identify research gaps and challenges facing the development of Maintenance 4.0 technologies. The authors recommend future research to explore specific industrial sectors and investigate topics such as sustainable maintenance systems and new business models.

Explainable Artificial Intelligence (XAI) is another crucial topic in the scope of machine learning, computational intelligence, and Industry 4.0. One of the articles published in this Special Issue addresses a specific XAI-related problem in fault detection in air handling units [5]. The study proposes a framework that utilizes the SHAP method to explain the output of an XGBoost classifier for fault detection and diagnosis tasks. The framework improves the confidence of visual perception and fault diagnosis compared to the default explanations generated by SHAP and has been validated by actual HVAC engineers through a survey.

Meanwhile, in [6], the authors explored the challenge of facilitating technology transfer between academia and industry. They proposed a framework that enables the deployment of advanced control methods that have been validated in lab settings to real-life industrial processes. The effectiveness of the proposed solution is demonstrated through its successful application in a combined heat and power plant located in Tallinn, Estonia, where Model Predictive Control was used as the advanced control algorithm. The flexible framework can accommodate any other type of advanced control algorithm, depending on the specific industrial control problem.

In [7], the challenges of implementing computer vision solutions for vehicle detection and localization in an IoT environment were examined. The focus was on deploying convolutional neural networks on edge devices with limited resources and on overcoming the challenge of compressing NN models. The authors then proposed a method that allows one to choose the most efficient CNN model compression method. The approach was then successfully verified on datasets stemming from industrial partners of the project.

The authors of [8] also explored issues related to edge computing and computer vision. In their work, they proposed the use of a novel discrete atomic compression algorithm for compressing digital images. Compared to classical approaches, such as using the ZIP compression algorithm, this method provides multiple advantages and holds great promise for edge applications, including artificial intelligence and machine learning.

In [9], the authors presented an active control system to prevent malfunctions in gasket plate heat exchangers. A noteworthy aspect of their work is the validation of the proposed approach on a real-life test bench. This aligns with this Special Issue’s theme of technology transfer, as it brings the solution one step closer to actual implementation in an industrial setting. The article also addresses the theme of intelligent control.

Continuing with the theme of control systems for Industry 4.0, the authors of [10] use the performance portrait method (PPM) to design optimal and robust proportional–integral–derivative controllers with two degrees of freedom (2DoF PID) for a double-integrator plus dead-time (DIPDT) process model. The PPM is used to verify the pilot analytical design of the parallel 2DoF PID controller and to illustrate design efficiency by analyzing the effects of different loop parameters on changing the optimal processes iteratively. The article highlights the contributions of PPM as an intelligent method for controller tuning that mimics an expert with sufficient experience to select the most appropriate solution based on a database of known solutions.

The theme of control continues in [11]. The article is not directly associated with the central concepts of Industry 4.0. Nevertheless, its contents are linked to the corresponding problem stack concerned with developing and implementing advanced control algorithms for industrial applications. In the article, the authors propose a real-time feasible implementation of a high-order H2 regulator, based on an FPGA, to stabilize electron beam arrival time in linear accelerators. The proposed digital solution is shown to cover high repetition rates as used in the ELBE system with simulation and synthesis results, verified through a dedicated FPGA testbench.

The study [12] introduces the evolutionary field theorem of search agents and proposes the Evolutionary Field Optimization with Geometric Strategies (EFO-GS) algorithm to improve the quality evolutionary searches. The study also modifies the multiplicative neuron model to develop Power-Weighted Multiplicative (PWM) neural models, which can better represent polynomial nonlinearity and operate in different modes. The application of these techniques in an electronic nose application demonstrates the potential impact of using neuroevolutionary machine learning to fulfill the goals of Industry 4.0.

The published articles in this Special Issue are highly diverse, but all of them share a common theme, which is the problems related to Industry 4.0. These issues are addressed from the perspective of advanced modeling and control problems, digital twins, and other contemporary technologies. The contributions of these authors are incredibly valuable as they allow us to fully realize the objectives of the Fourth Industrial Revolution and how to pave the way towards the next one.
